# Dyscalculia from a developmental and differential perspective

**DOI:** 10.3389/fpsyg.2013.00516

**Published:** 2013-08-21

**Authors:** Liane Kaufmann, Michèle M. Mazzocco, Ann Dowker, Michael von Aster, Silke M. Göbel, Roland H. Grabner, Avishai Henik, Nancy C. Jordan, Annette D. Karmiloff-Smith, Karin Kucian, Orly Rubinsten, Denes Szucs, Ruth Shalev, Hans-Christoph Nuerk

**Affiliations:** ^1^Department of Psychiatry and Psychotherapy A, General HospitalHall in Tyrol, Austria; ^2^Institute of Child Development, University of MinnesotaMinneapolis, MN, USA; ^3^Department of Experimental Psychology, University of OxfordOxford, UK; ^4^Department for Child and Adolescent Psychiatry, German Red Cross HospitalsBerlin, Germany; ^5^Department for Clinical Psychology, University of PotsdamPotsdam, Germany; ^6^MR-Center, University Children's HospitalZürich, Switzerland; ^7^Department of Psychology, University of YorkYork, UK; ^8^Department of Psychology, Georg-August-University of GoettingenGoettingen, Germany; ^9^Department of Psychology, Faculty of Humanities and Social Sciences, Ben Gurion University of the NegevNegev, Israel; ^10^School of Education, University of DelawareNewark, DE, USA; ^11^Centre for Brain and Cognitive Development, Department of Psychological Sciences, Birkbeck, University of LondonLondon, UK; ^12^Edmond J. Safra Brain Research Center for the Study of Learning Disabilities, Department of Learning Disabilities, University of HaifaHaifa, Israel; ^13^Department of Psychology, Centre for Neuroscience in Education, University of CambridgeGreat Britain, UK; ^14^Neuropediatric Unit, Shaare Zedek Medical CenterJerusalem, Israel; ^15^Department of Psychology, University of TuebingenTuebingen, Germany; ^16^IWM-KMRC, Knowledge Media Research CenterTuebingen, Germany; ^17^LEAD Graduate SchoolTuebingen, Germany

**Keywords:** developmental dyscalculia, developmental perspective, heterogeneity, individual differences, diagnosis, classification, research criteria

Developmental dyscalculia (DD) and its treatment are receiving increasing research attention. A *PsychInfo* search for peer-reviewed articles with *dyscalculia* as a title word reveals 31 papers published from 1991–2001, versus 74 papers published from 2002–2012. Still, these small counts reflect the paucity of research on DD compared to dyslexia, despite the prevalence of mathematical difficulties. In the UK, 22% of adults have mathematical difficulties sufficient to impose severe practical and occupational restrictions (Bynner and Parsons, 1997; National Center for Education Statistics, 2011). It is unlikely that all of these individuals with mathematical difficulties have DD, but criteria for defining and diagnosing dyscalculia remain ambiguous (Mazzocco and Myers, 2003). What is treated as DD in one study may be conceptualized as another form of mathematical impairment in another study. Furthermore, DD is frequently—but, we believe, mistakenly- considered a largely homogeneous disorder. Here we advocate a differential and developmental perspective on DD focused on identifying behavioral, cognitive, and neural sources of individual differences that contribute to our understanding of what DD *is* and what it is *not*.

## Heterogeneity is a feature of DD

DD is not synonymous with all forms of arithmetic and mathematical difficulties^1^. Here we emphasize that DD is characterized by severe arithmetic difficulties and accounts for only a subset of individuals with arithmetic difficulties [see Figure 2 in Kaufmann and von Aster (2012)]. In studies including children with various manifestations of arithmetic difficulties, true deficits of DD are likely to be masked because DD represents only a minority of children in these samples (Murphy et al., 2007; LeFevre et al., 2010). Any theory of DD must account for differences between DD and individual differences in arithmetic in the general population. Kaufmann and Nuerk (2005) claimed that, “… average arithmetic development does not pursue a straight, fully predictable course of acquisition, but rather can be characterized by quite impressive individual differences” (Siegler, 1995; Dowker, 2005). Arithmetic ability consists of many components [e.g., memorizing facts, executing procedures, understanding, and using arithmetical principles (Desoete et al., 2004; Dowker, 2005, 2008)], each subject to individual differences that continue into adulthood (Dowker, 2005; Kaufmann et al., 2011a) and may contribute to the reported prevalence of low numeracy (Geary et al., 2013). These individual differences must be considered when defining DD, because assumptions about a single core deficit (e.g., Butterworth, 2005) do not support the range of clinical manifestations of DD.

Moreover, heterogeneity of DD and other mathematics difficulties is also fostered by environmental factors, ranging from cultural factors (e.g., nature and extent of schooling, characteristics of the counting system) to the effects of pre-/postnatal illness or socio-emotional adversity (e.g., math anxiety). Hence, arithmetic difficulties may be associated with other learning disorders (i.e., dyslexia) or with various neuropsychiatric and pediatric disorders (e.g., attention-deficit hyperactivity-disorder/ADHD, epilepsy; Shalev and Gross-Tsur, 1993; Marzocchi et al., 2002; Kaufmann and Nuerk, 2008). Disentangling these types of arithmetic difficulties may be important given recent evidence that treating an underlying medical condition (i.e., attention disorder) may alleviate the arithmetic difficulties (Rubinsten et al., 2008).

Below, we emphasize the need for a developmental view on DD and suggest definitional criteria acknowledging its developmental nature, heterogeneous manifestations and distinctness from other forms of arithmetic/mathematical difficulties.

## Towards a developmental perspective on DD

**Figure 1:**
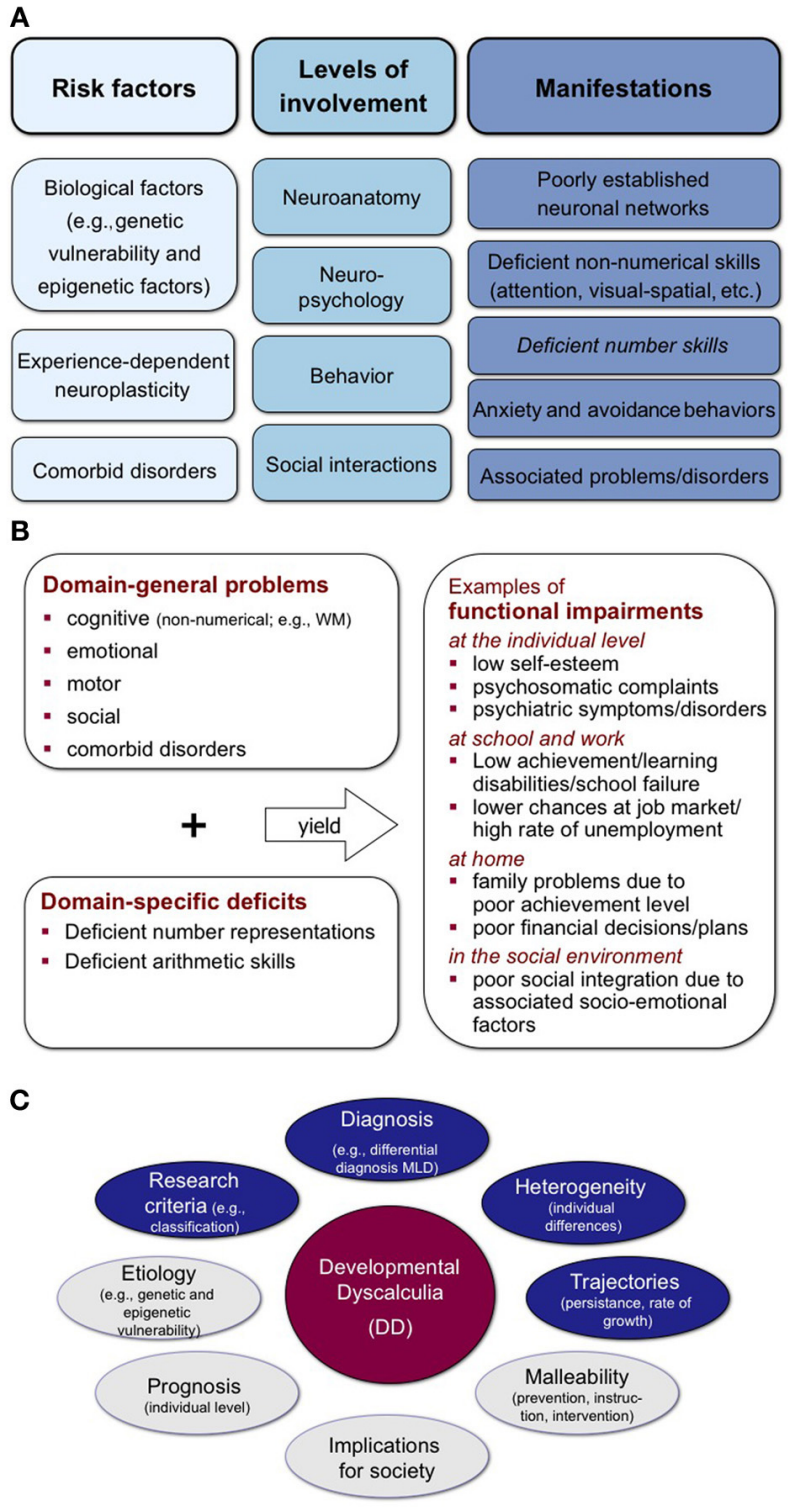
**(A)** A development and integrative perspective on DD. **(B)** Schematic representation of potential clinical manifestations of DD. **(C)** Schematic representation of key areas for future research endeavors targeted at elaborating true development conceptualizations of DD. Please note that topics written in gray ellipses are not the focus of the present paper, but are nevertheless important issues that await further systematic investigations.

A developmental perspective enables us to trace pathways of parallel and/or sequential mechanisms at varying processing levels (neuroanatomical, neuropsychological, behavioral, interactional; Figure 1A). Important questions facing researchers include whether DD represents the extreme end of a continuum (or several continua) of mathematical ability or whether the arithmetic difficulties associated with DD are qualitatively different from more common mathematics difficulties. There is evidence to support each of these positions.

Arithmetic difficulties can reflect individual differences in both numerical and non-numerical functions. The numerical functions comprise many aspects of “number sense” such as spontaneous focusing on number (Hannula et al., 2010), comparing numerical quantities represented non-symbolically (e.g., as dot arrays; Piazza et al., 2010; Halberda et al., 2012), processing numbers symbolically (e.g., in Arabic notation; Stock et al., 2010), or linking non-symbolic representations to symbols such as number words and Arabic numerals (Rubinsten et al., 2002; Rubinsten and Henik, 2005; Bugden and Ansari, 2011). These individual differences in “number sense” may reflect variation in neural pathways involved in even quite rudimentary aspects of numerical cognition (e.g., single digit arithmetic: Price et al., 2013). Studies of functional activation during magnitude comparison reflect developmental variations over time (for respective meta-analyses, see Houdé et al., 2010; Kaufmann et al., 2011b) and suggest variation in development *per se* rather than in comparable but delayed trajectories (Vogel and Ansari, 2012; Price et al., 2013).

Recently, Moeller et al. (2012) distinguished the following approaches: (i) DD is related to a numerical core deficit, (ii) DD subtypes exist due to domain-general processes, and (iii) DD subtypes exist due to domain-specific numerical deficits beyond the aforementioned core numerical deficit. The *core deficit hypothesis* assumes that DD is a coherent syndrome mainly linked to neurofunctional peculiarities of the intraparietal sulcus (Butterworth, 2005). However, the heterogeneous clinical picture of DD (Figure 1B) is at odds with a single core deficit assumption (Mazzocco, 2007; Rubinsten and Henik, 2009). The second approach suggests that different subtypes can be distinguished on the basis of associated *domain-general deficits*. For instance, deficits in verbal (working) memory, semantic memory or visual-spatial skills (Rourke and Conway, 1997; von Aster, 2000; Geary, 2004) and even in belief-laden logical reasoning (Morsanyi et al., 2013) reportedly influence arithmetic difficulties (although some results contradict any view of simple relationships between verbal/spatial discrepancies and arithmetical components; Dowker, 1998). Respective developmental calculation models acknowledging non-numerical influences have been proposed previously (von Aster and Shalev, 2007; Kaufmann et al., 2011b). Such domain-general cognitive deficits may account for individual differences in the clinical picture despite comparable core numerical deficits. Finally, *domain-specific numerical deficits* (Wilson and Dehaene, 2007) may reflect multiple and distinct genuinely numerical deficits specifically affecting magnitude representation, verbal number representations, arithmetic fact knowledge, visual-spatial number forms, ordinality, base-10-system, or finger representations of numbers (Temple, 1991; Mazzocco et al., 2011; Moeller et al., 2012).

## Current challenges related to DD classification, diagnosis, and research criteria

These aforementioned theoretical assumptions have important consequences for DD diagnosis and research. If, for instance, some children have severe problems in arithmetic fact retrieval but perform adequately on other numerical and arithmetic assessment tasks, they might not be classified as dyscalculic or even arithmetically impaired when assessments rely on a composite score comprising different numerical and arithmetic tasks. Deficits in one or few subsets that do not qualify for a DD diagnosis may still constitute severe problems for those children. In research designs, such delineated deficits might be undetected by group studies because averaging across participants and processes may mask deficits displayed by minorities (Siegler, 1987). The opposite risk also exists: children may be labeled, by themselves or others, as weak at arithmetic based on a specified difficulty despite average or high ability in other areas of arithmetic. This may lead to self-fulfilling prophecies or contribute to significant mathematics anxiety. Indeed, among young children, most studies suggest relatively little relationship between anxiety and performance, while in older children and adults, the relationship is strong and bidirectional; anxiety affects performance, and poor performance leads to anxiety (e.g., Ashcraft and Kirk, 2001; Mazzone et al., 2007; Pixner and Kaufmann, 2013).

Another major challenge of research on DD is the extensive range seen in diagnostic criteria and assessment tools used, which may influence research results (Murphy et al., 2007; Moser Opitz and Ramseier, 2012; Devine et al., 2013). As discussed by Moeller et al. (2012), there is little agreement about which children belong in the target group (DD, mathematical learning disability, etc.). Methodological approaches vary in terms of the cut-off points for classification criteria (ranging from <10 to <35 percentiles), whether reported percentiles reflect standardized or sample-based rankings, or deviations based on the population means and SDs. When different approaches are used across studies, very different children are included in study samples, and thus different background characteristics may be controlled for. Even children with general cognitive deficits may be included if a significant discrepancy between average intellectual abilities and sub-average math skills is not required as definitional criterion (as requested by the current Diagnostic and Statistical Manual of Mental Disorders (DSM) (Ehlert et al., 2012).

A final major challenge concerns the actual differential diagnostic classification tasks used in studies examining DD. While some studies employ discrete numerical tasks (e.g., dot enumeration), other studies use standardized math tests that may involve logical reasoning or text comprehension. Hence, apparently contradictory results as to whether DD involves deficits in basic or more complex numerical abilities may stem from the use of different classification tasks across studies. Discrepant findings may also reflect different samples of children who are nevertheless all presumed to have DD. The need is for research on DD to be both comprehensive and comparable across studies, which calls for a consortium-based proposal to adopt international standard diagnostic tools that are comparable across countries, curricula and therefore studies, in addition to study-specific assessments (as applicable).

## How developmental conceptualizations of DD may guide educational and therapeutic approaches

Beyond its scientific value, developmental conceptualizations of DD are crucial in guiding effective educational and therapeutic strategies. Researchers must consider the utility and meaningfulness of their contributions to the public perception of DD (including perceptions of teachers and parents). For instance, neurodevelopmental disorders like DD are at least partially attributable to inherited genetic differences (Shalev et al., 2001; Kovas et al., 2007). Hence, when conceptualized as a homogeneous and inborn disorder, DD may be misinterpreted as immune to the effects of behavioral interventions. A developmental approach considers multiple factors interacting to contribute to manifestations of DD. Such an approach is adopted in the forthcoming DSM-V, which replaces the categorical DSM-IV definition of distinct learning disorders (reading/written expression/mathematics) with an overarching multi-dimensional diagnosis of “Specific Learning Disorders” that acknowledges distinct manifestations of learning difficulties in various academic domains. However, in the theoretical debate about domain-specific versus domain-general underpinnings of DD, it is important to recall that domain-general deficits early on in development may result in seemingly domain-specific deficits in later development, because the earlier deficits may be more relevant to the computational demands of one domain (e.g., number) while still affecting other domains albeit to a more subtle degree. The reverse may also be true: numerical deficits may manifest as domain general deficits in, for instance, attention or working memory when diagnostic tools draw on numerical stimuli.

While advocating a developmental and differential perspective on DD, we must also caution against over-relying on adult neuropsychological patients with acquired mathematics disorders as models of DD (Kaufmann and Nuerk, 2005; Ansari, 2010; Karmiloff-Smith et al., 2012). As Karmiloff-Smith (1998) explains, important differences exist between deficits that arise during development versus those resulting from damage to an existing system. Therefore, we argue that (i) DD is a heterogeneous disorder resulting from individual differences in development or function at neuroanatomical, neuropsychological, behavioral, and interactional levels (Figure 1A), and that (ii) an understanding of these differences can facilitate DD diagnosis and intervention. The acknowledgement of individual differences characterizing DD calls for adequate methodological and differential diagnostic approaches, and adequate attention to the developmental component of DD (reflecting systematic inter- and intra-individual variations between age and skill levels) (Figure 1C). Solid developmental conceptualizations of DD may foster the acceptance of DD as a disorder and raise public awareness for the need to provide targeted educational, therapeutic, and structural support tailored to affected individuals (Figure 1B), as well as differentiating DD from other sources of difficulty in children underperforming in mathematics.

As a synopsis of our arguments, we propose the following preliminary definition of DD:

***Primary DD** is a heterogeneous disorder resulting from individual deficits in numerical or arithmetic functioning at behavioral, cognitive/neuropsychological and neuronal levels. The term **secondary DD** should be used if numerical/arithmetic dysfunctions are entirely caused by non-numerical impairments (e.g., attention disorders*)^2^.

Further, we postulate the following recommendations for primary DD (and its diagnosis):

- There is convincing evidence that basic numerical skills are impaired in DD. Therefore, purely educational (curricular) tests are not adequate to tap the characteristic numerical deficits associated with DD.
- DD is a heterogeneous disorder (like other neurodevelopmental disorders). Multi-dimensional assessments tracking different numerical representations and arithmetic processes should be used to evaluate response accuracy, speed, and strategies.
- Specific deficits in numerical subdomains are possible, even when overall dyscalculia test scores are unremarkable.
- Arithmetic performance of children diagnosed with DD can be unstable over development and time; thus children who are reasonably close to formal DD criteria (usually scoring <10th percentile) should be retested within the following school semester/year. Conservatively, retesting is recommended if performance is <25th percentile.
- Currently, there is no evidence that focusing on discrepancies between numerical and general cognitive skills improves diagnostic accuracy or interventional outcomes.
- DD can be comorbid with other neurodevelopmental, psychiatric, and neuropediatric disorders that may affect the regulation of motor/executive/affective/socio-behavioral functioning and have to be considered for differential diagnosis.
- Educational and socio-emotional characteristics should be considered in diagnosing and ruling out DD.

## Footnotes

^1^The terms “arithmetic” and “mathematical” are not synonymous as the former refers to computational skills (i.e., processing of basic arithmetical operations such as addition/subtraction/multiplication) and the latter encompasses other aspects of numerical thinking such as algebra, geometry, etc.

^2^Likewise, Geary (2007) distinguished primary and secondary biological routes to math learning disabilities.
